# Postprandial Antioxidative Response to Ingestion of Formulated Date- and Fruit-Based Nutritional Bars by Healthy Individuals

**DOI:** 10.3390/nu16111794

**Published:** 2024-06-06

**Authors:** Manahel A. Alolyan, Hani A. Alfheeaid, Ahmad H. Alhowail, Majed M. Alamri, Modhi S. Alghasham, Nada A. Alzunaidy, Hassan Barakat

**Affiliations:** 1 Department of Food Science and Human Nutrition, College of Agriculture and Food, Qassim University, Buraydah 51452, Saudi Arabia; 431214354@qu.edu.sa (M.A.A.); h.alfheeaid@qu.edu.sa (H.A.A.); n.alznedy@qu.edu.sa (N.A.A.); 2 Department of Pharmacology and Toxicology, College of Pharmacy, Qassim University, Buraydah 51452, Saudi Arabia; 3 Department of Laboratory and Blood Bank, Qassim University Medical City, Buraydah 51452, Saudi Arabia; mamri@qumc.edu.sa; 4 Department of Obstetrics and Gynaecology, Qassim University Medical City, Buraydah 51452, Saudi Arabia; mghasham@qumc.edu.sa; 5 Food Technology Department, Faculty of Agriculture, Benha University, Moshtohor 13736, Egypt

**Keywords:** postprandial antioxidants, oxidative stress, health, food supply, nutrition, diet

## Abstract

Nutritional bars (NBs) are gaining popularity among healthy and athletic individuals, but postprandial antioxidative response has not been investigated. Therefore, the current study examined the postprandial alterations in total phenolic content (TPC), total antioxidant capacity (T-AOC), malondialdehyde (MDA), and Superoxide dismutase (SOD) in the plasma of healthy individuals after the ingestion of 140 g (510 Kcal) from formulated date-based bars (DBBs) or fruit-based bars (FBBs). Firstly, the free and bound phenolic contents (PCs) were determined to be 10.15 and 12.98 and 6.19 and 3.57 mg GAE g^−1^, respectively. FBBs were significantly higher in free PC than DBBs, while DBBs were considerably higher in bound PC than FBBs. Secondly, twenty participants with age, height, weight, body mass index (BMI), fat mass, and fat-free mass averages of 21.4 years, 170.0 cm, 66.3 kg, 22.9 kg m^2^, 14.5, and 29.2 kg, respectively, were subjected to metabolic experiments (ISRCTN19386758). Ingestion of 140 g of FBB or DBB resulted in 288.50 or 302.14 µg TPC mL^−1^ blood, respectively. Postprandial TPC content increased with time progression and peaked after 120 min. T-AOC contents averaged 22.63 and 23.61 U mL^−1^ before ingestion of FBBs or DBBs, respectively. The T-AOC content increased significantly 120 and 180 min after ingestion of DBBs, while no significant change was noted after consuming FBBs. A significant decrease in MDA content was observed 180 min after consuming DBBs, while no significant change was noted after consuming FBBs. SOD concentrations ranged from 193.99 to 201.07 U L^−1^ in FBBs and DBBs, respectively. No considerable response was noted up to 3 h after ingestion of FBBs. On the contrary, a significant response was found 120 min after consuming DBBs. Pearson’s correlation coefficient indicated a highly significant positive correlation coefficient (*p* < 0.01) between T-AOC and either MDA or SOD, as well as between MDA and SOD. The principal component analysis demonstrated a strong and positive relationship between SOD and TPC at 60 and 120 min after DBB ingestion. In conclusion, the relative changes in postprandial responses in T-AOC and MDA did not significantly (*p* > 0.05) differ between DBBs and FBBs, except for TPC (*p* = 0.04, paired *t*-test) and SOD (*p* = 0.003, paired *t*-test). Further studies with an extended experimental time are needed to confirm the current findings.

## 1. Introduction

The overproduction of reactive oxygen species (ROSs) due to an inadequate endogenous antioxidant defense mechanism is known as oxidative stress [1]. Oxidative stress damages lipids, proteins, and DNA, increasing the risk of chronic diseases like cardiovascular disease, cancer, chronic kidney disease, and osteoporosis [2,3]. Several biomarkers, including total phenolic content (TPC), malonaldehyde (MDA), and others, can be used to assess the levels of oxidative stress in the body [4], total antioxidant capacity (T-AOC) [5], and superoxidase dismutase (SOD) [6,7]. The impact of diverse diets on human oxidative stress has been the subject of numerous intriguing investigations [8,9]. According to the available evidence, a high-fat, high-protein, and carbohydrate diet is associated with increased oxidative stress [9,10]. On the contrary, consuming foods that are rich in antioxidants, such as fruits and vegetables, or taking antioxidant supplements might help lower oxidative stress levels in the body [11,12,13].

Forecasts indicate that the snack bar industry will grow from $15 billion in 2019 to $19 billion in 2025 [14]. The size of the energy bar market was 682.26 million USD in 2022 and is predicted to reach 1131.08 million USD by 2030, increasing at a Compound Annual Growth Rate (CAGR) of 5.78% from 2023 to 2030 [15]. The energy bar market will be driven by health complexity and health consciousness in the coming years. Growing demand for energy snacks, drinks, and other items drives the industry. Due to their high nutrient content, ready-to-eat nature, permanent availability, and inexpensive cost, nutritional supplements have garnered attention recently. Recently, people have been using dietary supplements, including fruit bars made from diverse fruits [16,17]. Functional nutrition bars may be an alternative delivery format for supplements to help support overall metabolic health for the general population and athletes. Supplemental functional nutrition bars may promote overall health by containing higher protein, fiber, antioxidants, and essential vitamins and minerals than conventional commercial bars. Fruit-based snack bars meet nutritional needs with natural sugars, vitamins, minerals, and bio-nutrients [18]. Date fruit-based snack bars can benefit from dates’ high nutritional content, which includes dietary fibers, unsaturated fatty acids, and a wide range of micronutrients and bioactive compounds [19]. Fruit-based snack bars are commonly utilized to meet consumers’ daily nutritional needs by incorporating natural sugars, vitamins, minerals, and other bio-nutritive components [18]. Additionally, several research studies have shown that making different DBBs can be profitable due to their nutritional and functional benefits [17,20,21]. DBBs can meet domestic and worldwide consumer demand by providing macro- and micronutrients, dietary fiber, and bioactive compounds [22] and are expected to achieve greater international marketability [23]. Dates are rich in phytochemicals, including carotenoids and phenolic compounds [24]. Fresh and dried dates and different types of dates can have vastly varying total phenolic contents. Ferulic, hydroxybenzoic, gallic, caffeic, vanillic, chlorogenic, isovanillic, protocatechuic, isoferulic, and hydroxycinnamic acids were discovered in Ajwa dates [25]. Other dates, like Sukkari, Mabroom, Khalas, and Nabtat-Saif, contain flavonoids such as quercetin, luteolin, apigenin, isoquercitrin, and rutin [26]. These phenolic compounds reduce lipid peroxidation, reduce oxidative stress, and improve antioxidant capabilities [24].

In toxicant-treated rats, date extracts significantly increased antioxidant indicators such as glutathione transferase, catalase, glutathione reductase, and MDA [27,28,29,30]. A recent double-blind randomized controlled research study found that date seed powder improved oxidative stress markers such as SOD, glutathione peroxidase, and MDA [31]. Gutierrez-Mariscal et al. [32] used Coenzyme Q10 (CoQ), an antioxidant that affects oxidative stress. CoQ modifies p53 activation/stabilization in older people to boost their antioxidant capabilities. The saturated fatty acid-rich diet had higher postprandial 8-OHdG, p53 mRNA, and monoubiquitinated p53 and lower Mdm2 mRNA than Mediterranean (Med) and Med + CoQ diets. Cellular oxidative processes were reduced, and the Med + CoQ diet prevented aging. Postprandial cytoplasmatic p53, nuclear p-p53 (Ser20), and monoubiquitinated p53 protein decreased after the Med + CoQ diet. The Med + CoQ diet decreased cellular oxidation and improved oxidative DNA damage in aged people. Antioxidants minimize oxidative modification of LDL cholesterol, block spontaneous glucose oxidation, boost NOx bioactivity, and reduce postprandial degradation [33]. Huang et al. [34] noted that when fasting times increased, postprandial MDA, T-AOC, and SOD levels dropped, while plasma fluorescent products (FlOPs) remained unaffected. In the same context, tahini may benefit healthy people’s metabolic and antioxidant status biomarkers; thus, dietitians and other health professionals may recommend adding it to our daily diets to promote healthy diet consumption [35]. One study found that berry-based drinks reduce postprandial oxidative stress. The data showed that berry concentrate inhibits stomach lipoperoxidation processes, mainly during the thermal treatment of foods [36]. It should be mentioned that consuming foods that are rich in antioxidants can help prevent several diseases and issues, especially in those who are overweight [37]. Zhang et al. [38] stated that water caltrop husk controlled blood glucose and improved the status of antioxidants in diabetes.

Insufficient reports have assessed the body’s antioxidant response upon consuming regular diets. For instance, innovatively designed DBBs and FBBs [39] were produced, and metabolic trials were carried out. It was found that the energy and macronutrient composition of the formulated DBBs and FBBs were identical, but their types of sugars, fiber, micronutrients, and phytochemicals differed. Therefore, the current study aims to compare postprandial changes in oxidative stress in response to consumption of DBBs and FBBs [39] and their impact on antioxidant status. To achieve this, antioxidative activity was evaluated using the TPC, T-AOC, MDA, and SOD concentrations in the plasma of healthy adult volunteers in a fasted state and three hours after ingestion of both DBBs and FBBs.

## 2. Materials and Methods

### 2.1. Formulation of DBBs and FBBs

The investigated nutritional bars were made from either a fruit-mixture-based bar (FBB) or a Sukkari date-based bar (DBB) with a variety of date palm “*Phoenix dactylifera* L.” as a base for both bars. The proximate composition, mineral and vitamin content, sugar profiles, visual color, and amino and fatty acid profiles of DBBs and FBBs were comparatively analyzed and previously published [39].

### 2.2. Phytochemical Analysis of DBBs and FBBs

The total phenolic compounds (TPCs) in the DBBs and the FBBs were estimated using the Folin–Ciocalteu method, according to Bettaieb et al. [40]. Briefly, 2 g of the sample was extracted in 10 mL of 70% methanolic aqueous solution three times and then centrifuged at 10,000× *g* for 10 min at 4 °C. Afterwards, 50 µL of the clear supernatant was mixed with 100 µL of Folin–Ciocalteu reagent for 5 min. Then, 150 µL of an alkali solution (7.5% Na_2_CO_3_) was added. The mixture was incubated in the dark for 60 min at 23 °C. Absorbance was then measured at 765 nm using a microplate reader (BioTek, Winooski, VT, USA). TPC was then expressed as milligram gallic acid equivalents per gram bar (mg GAE 100 g^−1^ dw). Free and bound phenolic compounds (PCs) were determined as described by Krygier et al. [41]. Briefly, 5 g of DBB or FBB was defatted with hexane and then dissolved in 100 mL of methanol–acetone–water (7:7:6, *v*:*v*:*v*) and ultrasonicated for 20 min at 30 °C. Afterward, the sample mixture was centrifuged at 4000× *g* for 5 min, and both clear supernatants and residue were collected after 3 extraction times. The clear supernatant was evaporated at 40 °C, adjusted to pH 2, extracted again with diethyl ether–ethyl acetate (1:1, *v*:*v*) 4 times, evaporated, and redissolved in 25 mL 80% methanol, and then the free PC was determined following the procedure of Bettaieb et al. [40]. For the determination of bound PC, the residue was initially dispersed in 25 mL of 4 M NaOH, stirred for 4 h, acidified to pH 2, and centrifuged. The clear supernatant was evaporated at 40 °C, reextracted with diethyl ether–ethyl acetate (1:1, *v*:*v*) 4 times, evaporated, and redissolved in 25 mL 80% methanol, and then bound PC was determined following the procedure of Bettaieb et al. [40].

### 2.3. Participants

Twenty healthy male participants aged 19–25 with a body mass index (BMI) of <25 kg m^−2^ and who were non-smokers, were not taking any medications or dietary supplements, and had had a stable body weight for at least four months before enrollment in the study were recruited. The study participants were recruited through advertising leaflets and verbal invitations at the Qassim University main campus in Saudi Arabia. Each potential participant underwent a health screening check, measuring their body weight and height. The study excluded any participant who suffered from eating disorders or chronic illness, ever had gastrointestinal surgery, or did not meet the study inclusion criteria. Written informed consent was obtained from each eligible participant before starting the study’s experimental trials. The study was approved by the Research Ethics Committee, Deanship of Scientific Research, Qassim University (Approval No. 24-74-04). This clinical trial was registered in the ISRCTN.com registry (ISRCTN19386758).

### 2.4. Anthropometric Measurements

Height was measured to the nearest 0.1 cm using a distance scale (Seca, Leicester, UK). Body weight and composition, including muscle mass, fat mass, and body mass index (kg m^−2^), were measured using the InBody 370S bioelectrical impedance analyzer (BIA) (InBody Co., Seoul, Republic of Korea).

### 2.5. Study Design and Experimental Trails

This was a randomized cross-over study where participants were asked to conduct two experimental trials, consuming a DBB on one day and an FBB on the other day, and these were separated by 5 to 7 days in between. The participants reported to the Nutrition and Metabolic Investigation Unit at Qassim University between 8:00 and 9:00 A.M. in a fasting state. A nurse specialist inserted a venous cannula, and a 4 mL fasting blood sample was subsequently collected in an EDTA tube (Jiangsu Kangyou Medical Instrument Co., Ltd., Changzhou, China). Following this, according to the assigned trial type, participants were asked to consume 140 g (containing about 510 kcal) of either a DBB or an FBB. After ingesting the trial bars, blood samples were collected at 60, 120, and 180 min. The blood samples were centrifuged at 3000× *g* rpm for 15 min to separate plasma and stored in a −80 °C ultra-freezer until analysis. During the experimental trials, food intake was prohibited, but water was provided ad libitum.

### 2.6. Antioxidant Biomarkers in Blood Samples

#### 2.6.1. Plasma Total Phenolic Content

The plasma TPC was estimated using the Folin–Ciocalteu reagent, according to Serafini et al. [42], with minor modifications. Briefly, 100 µL of fresh blood plasma was mixed gently with 300 µL of 10% Tri-chloroacetic acid aqueous solution, vortexed for 1 min, and centrifuged at 10,000× *g* for 10 min. A total of 100 microliters of clear supernatant was mixed with 100 µL Folin–Ciocalteu reagent (1:10 reagent–water), and 100 µL of 7.5% NaCO_3_ aqueous solution was added after 5 min. After incubation for 60 min in dark conditions, the absorbance of the formed color was measured spectrophotometrically using an Epoch microplate reader (BioTek Instruments, Winooski, VT, USA) at 725 nm. A standard curve was plotted using gallic acid (R^2^ = 0.99), and results were expressed as gallic acid equivalents per mL blood plasma (μg GAE mL^−1^ plasma).

#### 2.6.2. Levels of T-AOC, MDA, and SOD in Plasma

The levels of T-AOC (U L^−1^) in blood plasma were determined using a human T-AOC ELISA-based kit (No. E2199Hu), levels of MDA (nmol mL^−1^) were measured using a human MDA ELISA-based kit (No. E1371Hu), and levels of SOD (U L^−1^) were examined using a human SOD ELISA-based kit (No. E0918Hu). According to the kit instructions, the levels of the mentioned antioxidant biomarkers were measured at 450 nm using an Epoch microplate reader (BioTek Instruments, Winooski, VT, USA).

### 2.7. Statistical Analysis

Statistical analysis was performed using SPSS (ver. 22.0 for Windows, IBM, Houston, TX, USA). Statistical significance was tested with repeated two-way ANOVA followed by a post hoc test, and a *p*-value < 0.05 was applied, according to Steel et al. [43]. Experimental results were expressed as mean ± SE. Pearson’s correlation analysis was estimated, and the obtained correlation results were compared to critical values of Pearson’s *r* table under significance levels with a two-tailed test. Moreover, principal component analysis was also carried out.

## 3. Results

### 3.1. Free and Bound Phenolic Contents in DBBs and FBBs

The free PC and bound PC were determined in the formulated DBBs and FBBs, as shown in Table 1. The free PC was significantly higher in FBBs than in DBBs. On the other hand, DBBs contained considerably higher amounts of bound PC than FBBs.

### 3.2. Characteristics of the Study Participants

The average age, height, weight, and BMI of the study participants were 21.4 ± 0.9 years, 170.0 ± 1.3 cm, 66.3 ± 1.7 kg, and 22.9 ± 0.4 kg m^−2^, respectively (Table 2). The determined body compositions exuded 14.5 ± 1.1, 29.2 ± 0.9, and 37.9 ± 0.9 kg for fat mass, fat-free mass, and total body water, respectively.

### 3.3. Responses of Antioxidant Biomarkers in the Blood of the Study Participants

**Figure 1:**
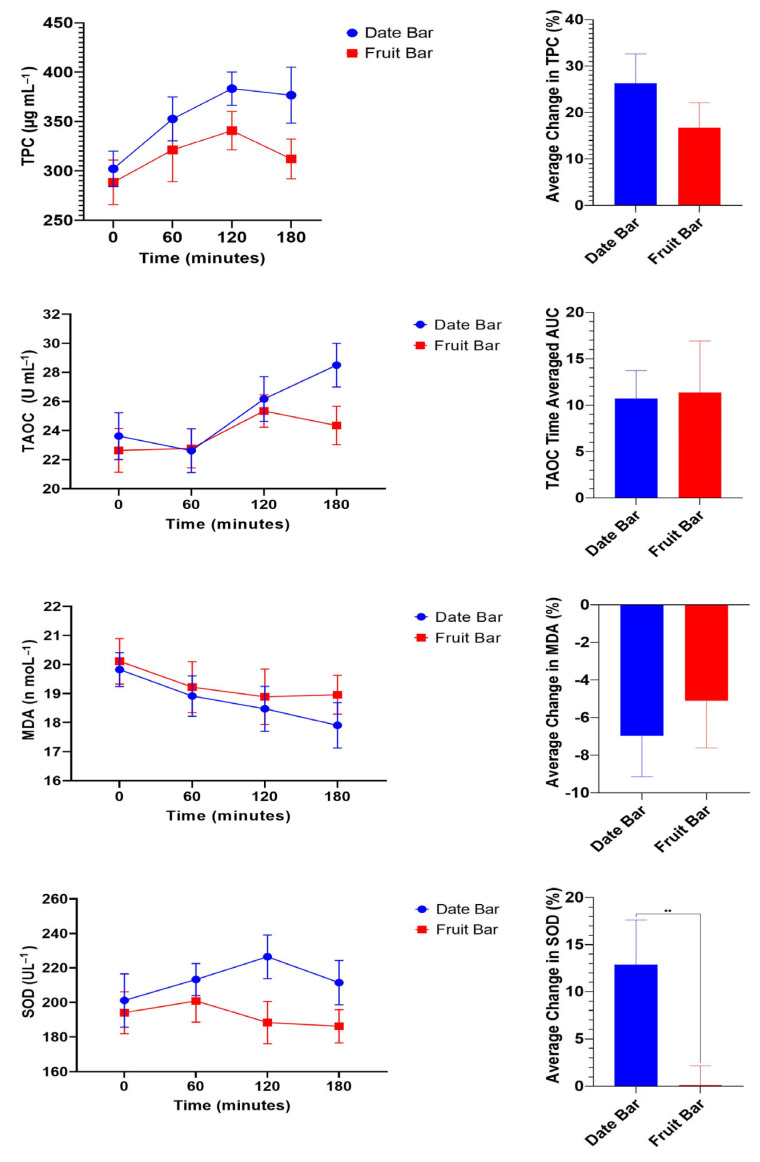
Postprandial responses of plasma concentrations of TPC, TOAC, MDA, and SOD. The antioxidant plasma concentrations were not significantly different (*p* > 0.05, trial effects, two-way ANOVA) between the DBB and FBB trials (*n* = 20). Data were further analyzed to investigate relative changes in antioxidant plasma concentrations, which were calculated as percentage increases or decreases in plasma concentrations from baseline (fasting) measurements. The results showed that DBB ingestion led to a significant (*p* = 0.006, paired *t*-test) relative increase in SOD plasma concentrations (mean difference: 12.7 ± 4 SEM) compared to FBBs. Relative TPC, TOAC, or MDA changes did not differ significantly (*p* > 0.05) between the two bars. Three hours of AUC values were calculated for antioxidant plasma concentrations following ingestion of the DBBs and FBBs. AUC was calculated using a trapezoid approach [44] to compare the different effects of DBBs and FBBs on plasma concentrations of antioxidants. These were significant only for TPC (*p* = 0.04, paired *t*-test) and SOD (*p* = 0.003, paired *t*-test) plasma concentrations but not for TAOC or MDA. “**”: indicate high significance (*p* < 0.01).

Postprandial changes in plasma TPC are shown in Figure 1. Ingestion of 140 g of FBB or DBB resulted in TPC in the blood within a range of 288.50 ± 22.57 to 302.14 ± 17.92 µg mL^−1^. Levels of TPC increased with the extension of time after the consumption of either FBBs or DBBs. The postprandial measurements (60, 120, and 180 min.) showed significant changes in plasma TPC, reaching their highest levels at 120 min following the ingestion of the bars. DBBs showed higher TPC content in the plasma compared to FBBs. No significant difference was noted between the trials at 60 min following ingestion of the FBBs and DBBs, while a significant difference was noted after 120 min and until 180 min.

Similarly, T-AOC changes in the plasma after consuming FBBs or DBBs were monitored, and the data are plotted in Figure 1. The T-AOC content averaged 22.63 ± 1.50 and 23.61 ± 1.61 U mL^−1^ for participants before ingestion of both bars, respectively. A significant increase in T-AOC content was observed 120 min after consuming DBBs, while no significant changes were noted following the ingestion of FBBs.

A significant difference was noted in DBB eaters after 180 min, whereas participants who consumed DBBs showed higher T-AOC content.

Oxidative stress is related to MDA concentration, as increasing antioxidant levels decrease the MDA content in plasma and tissues. The MDA concentration in the plasma was examined and is illustrated in Figure 1. The MDA concentrations in fasting blood samples ranged from 19.83 ± 0.58 to 20.11 ± 0.78 nmol mL^−1^ in the DBB and FBB groups, respectively. A significant decrease in MDA content was observed 180 min after consuming DBBs, while no significant change was noted after ingestion of FBBs. Comparing the DBB and FFB groups, no significant difference was found between consuming FBBs or DBBs. ROSs, an uncontrolled by-product of oxygen metabolism, can damage cells in numerous ways. Thus, SOD content was determined and is presented in Figure 1. SOD concentrations ranged from 193.99 ± 12.11 to 201.07 ± 15.46 U L^−1^ in the FBB and DBB groups, respectively. No significant response was noted up to 3 h after ingestion of FBBs. On the contrary, an effective response was found 120 min after consuming DBBs. Data were further analyzed to investigate relative changes in antioxidant plasma concentrations, which were calculated as percentage increases or decreases in plasma concentrations from baseline (fasting) measurements. The results showed that the date bar led to a significant (*p* = 0.006, paired *t*-test) relative increase in SOD plasma concentrations (mean difference: 12.77%) compared to the fruit bar. Relative TPC, TOAC, or MDA changes did not differ significantly (*p* > 0.05) between FBBs and DBBs. Subsequently, the three-hour area under curve (AUC) values were calculated for all oxidative stress biomarkers following the ingestion of FBBs or DBBs. These were significant only for TPC (*p* = 0.04, paired *t*-test) and SOD (*p* = 0.003, paired *t*-test) plasma concentrations but not for TAOC or MDA.

### 3.4. Statistical Correlation Coefficients among Oxidative Stress Biomarkers

The Pearson’s correlation coefficients of postprandial oxidative stress biomarkers in the blood of selected healthy individuals upon consuming either FBBs or DBBs were statistically calculated using the SPSS program and are tabulated in Table 3. A significant positive correlation coefficient (*p* < 0.05) between the time and TPC, a high significant positive correlation coefficient (*p* < 0.01) with T-AOC, a significant negative correlation coefficient (*p* < 0.05) with MDA, and no significant correlation coefficient with SOD were found. No significant correlation between the TPC and T-AOC, MDA, or SOD was recorded. On the contrary, a high significant positive correlation coefficient (*p* < 0.01) between T-AOC and either MDA or SOD was found. Additionally, a highly significant negative correlation coefficient (*p* < 0.01) between MDA and SOD was observed.

### 3.5. Principal Component Analysis (PCA)

**Figure 2:**
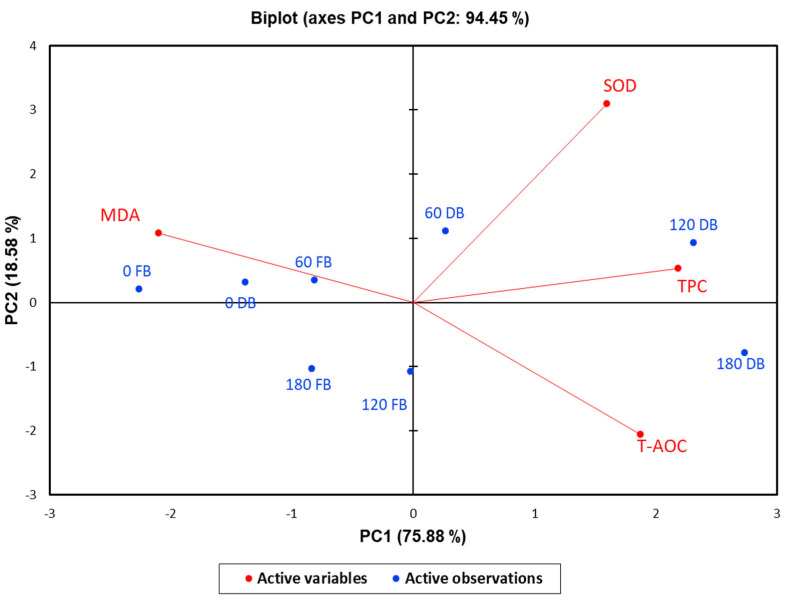
Principal component analysis for the postprandial responses of oxidative stress biomarkers upon ingestion of 140 g of formulated FBBs and DBBs.

**Figure 3:**
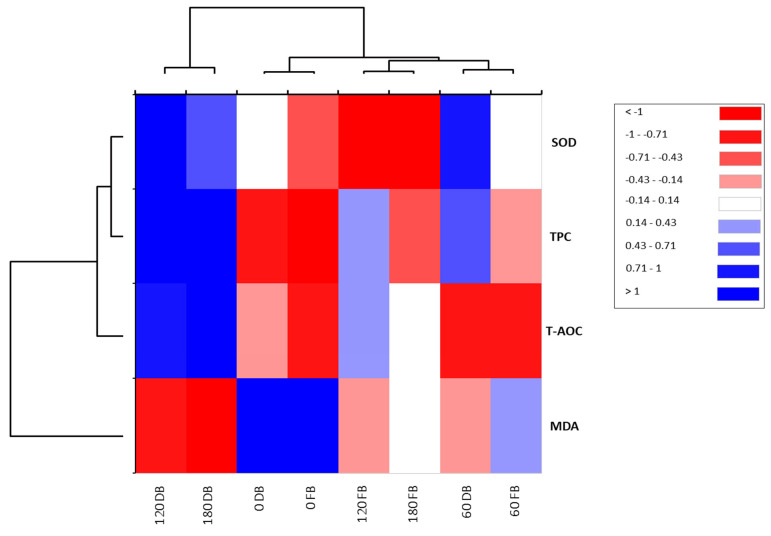
Heatmap for the postprandial responses of oxidative stress biomarkers upon ingesting 140 g of formulated FBBs and DBBs.

To estimate the correlations between postprandial responses to DBBs and FBBs on oxidative stress biomarkers such as TPC, T-AOC, MDA, and SOD, principal component analysis was conducted. A total of two components were selected (PCs 1 (75.88%) and 2 (18.58%)), which presented the most variability, with a total cumulative variance of 94.45% (Figure 2). PC1 displayed the uppermost variance, reaching 75.88%, whereas PC2 had 18.58%, reflecting a low variation. The analysis of blood samples at 60, 120, and 180 min after ingestion of DBBs was located on the positive side of PC1. Furthermore, a solid and positive relationship between SOD and TPC at 60 and 120 min after DBB ingestion was detected, while MAD was correlated negatively. Otherwise, the analyzed biomarkers at 0 time (fasting samples) from either DBBs or FBBs, in addition to at 60 and 180 min, were found on the opposite side of PC1, reflecting lower variation in response to these treatments. When a heatmap was applied, the times of the implemented sample analysis of both date bars (DBs) and fruit bars (FBs) were categorized into two clusters (Figure 2). The first cluster included two analysis times (120 and 180 min) for DBs, whereas the second had the analyzing times. A strong and positive correlation was observed among SOD, TPC, and TOAC in line with 120 and 180 min after the ingestion of DBBs. Moreover, SOD and TPC showed a positive and significant association within 60 min in the same treatment. The measured oxidative stress biomarkers were divided into two groups. The first group involved SOD, TPC, and T-AOC, while the second group consisted of MDA (Figure 3).

## 4. Discussion

Innovatively, our high-energy and high-protein bars based on Sukkari dates or a fruit mixture were formulated and comparatively analyzed [39]. In our previous study, proximate analysis showed that DBBs contained more ash and crude fiber than FBBs. In contrast, FBBs had higher moisture and fat content, with no differences in protein content. Dates and fruits changed DBB and FBB sugar profiles. In DBBs, Ca, Cu, Fe, Zn, Mn, and Se were more abundant than Mg, K, and Na. DBBs scored higher in lysine, methionine, histidine, threonine, phenylalanine, and isoleucine than FBBs. In contrast, FBBs scored higher in leucine and valine than DBBs. Palmitic acid dominated DBBs and FBBs. Most of the monounsaturated fat was oleic acid. Linoleic acid dominated the eight polyunsaturated fatty acids examined. Additionally, high α-linolenic acid concentrations were observed. The Sukkari DBB is nutrient-dense, applicable, convenient, economical, affordable, and a better sugar alternative that meets calorie needs [39]. The phytochemicals and antioxidant capacity of the DBBs and FBBs investigated in the past led to the conclusion that FBBs were significantly higher in free PC than DBBs. At the same time, DBBs were considerably higher in bound PC than FBBs, possibly due to the initial phenolic contents and matrix nature of the ingredients used. Date fruits contain many bioactive phytochemicals, like phenolic acids, polyphenols, and carotenoids [26,45,46,47], and, as shown in recent in vitro and in vivo investigations, have functional or pharmacological advantages [19]. Our most recent review compiled research on using date fruit for creating healthy snack bars [39]. DBBs were developed to enrich protein since dates are high in carbohydrates and low in proteins. The composition, absorption, bioavailability, and taste of food depend on its ingredients. These ingredients can deliver functional attributes, but novel ingredients like plant seeds and fruit polyphenol extracts can provide much higher qualities [13,48,49,50,51,52,53].

Interestingly, there has been a dearth of research on the effects of a regular diet on postprandial oxidative stress in humans [8,32,34,35,36,37,38]. Since bars are quick options for health-conscious individuals, it has become necessary to find studies investigating the postprandial responses of oxidative stress biomarkers in healthy individuals. Remarkably, no studies have investigated the postprandial oxidative stress changes in humans following the supplementation of functional nutrition bars. Thus, a metabolic experimental study concerning the postprandial alteration of TPC, T-AOC, MDA, and SOD in healthy individuals after consuming either FBBs or DBBs was undertaken. It was found that TPC, T-AOC, MDA, and SOD levels increased as the time after meal consumption progressed.

Levels of TPC significantly increased with the extension of time after consuming either FBBs or DBBs. The highest postprandial TPC content was reached 2 h after consuming the formulated bars. Similarly, Baxevanis et al. [35] showed a significant increase in total phenolic content from 0 to 4 h post-tahini ingestion, and similar studies have supported our findings [34,35,36,38]. Subsequently, the 3 h AUC value was significant for TPC plasma concentrations (*p* = 0.04, paired *t*-test), which confirms the postprandial change and bioavailability. Notably, in a postprandial study of sixteen volunteers, walnut consumption resulted in a 6.5% increase in plasma total phenolic content over 5 h, approaching statistical significance [54]. However, TPC content decreased from 180 min, as phenolics may be consumed as antioxidants to combat free radicals released during metabolism, as shown in [35].

Regarding T-AOC content, the postprandial response related to the consumed bars gradually increased after 1 h in associative correlation with the postprandial TPC content. The T-AOC content in the plasma of individuals who consumed the DBBs significantly increased up to 3 h, while no significant change was noted after consuming FBBs. This may be due to the date matrix, which gradually provides antioxidants with the progression of the digestion process. Similarly, a postprandial increase in T-AOC was observed between 0 and 2 h following consumption [34]. MDA is one of the products formed by the reaction of lipids with oxygen free radicals, and its level represents the level of lipid peroxidation. Oxidative stress is related to MDA concentration, as increasing antioxidants decreases the MDA content in plasma and tissues. MDA concentrations did not significantly decrease for up to 3 h when comparing DBBs and FFBs. Our study found that released TPC and increased T-AOC combatted radicals generated by the consumption of the bar during the monitored 0–3 h, lowering postprandial MDA levels. The results showed a further decrease in MDA levels as a postprandial response in associative correlation to digestion.

Interestingly, the 3 h AUC values for T-AOC and MDA plasma concentrations were insignificant when DBBs and FBBs were compared. Huang et al. [34] proved that they cause oxidative stress in humans, particularly damage to lipids through oxidation. Possible oxidation damage prevention and postprandial oxidative stress enhancement were used for the formulated bars [34,35,38]. Their findings also showed that MDA levels decreased during the first 2 h of fasting and increased after 2 to 4 h of regular consumption. The constant rise in lipid oxidation could account for the negligible shift in MDA levels between 0 and 3 h, and the antioxidants absorbed were not enough to make a difference [34]. Fisher-Wellman et al. [10] discovered that MDA peaked after 4 h of fasting on a lipid diet. Several studies have also seen decreased antioxidant levels from 1 h of fasting [55,56]. One study found that older women’s postprandial total antioxidant activity was increased even when they consumed foods with low levels of antioxidants [57].

ROSs are produced as a by-product of oxygen metabolism, and if not adequately controlled, it can cause various types of cell damage. SOD enzymes catalyze the dismutation of superoxide (O^2•−^), generating hydrogen peroxide (H_2_O_2_). SOD enzymes control the levels of a variety of ROSs and RNS, thus both limiting the potential toxicity of these molecules and controlling broad aspects of cellular life that are regulated by their signaling functions [58]. No significant response was noted up to 3 h after consuming FBBs. On the contrary, a powerful response was found 120 min after consuming DBBs. The results showed that the date bar led to a significant (*p* = 0.006, paired *t*-test) relative increase in SOD plasma concentrations (mean difference: 12.77%) compared to FBBs. The 3 h AUC values for SOD plasma concentrations were significant (*p* = 0.003, paired *t*-test). Huang et al. [34] correlated with our findings by showing an increase in T-AOC and SOD levels between 0 and 2 h. It could be explained by (i) the diet boosting the antioxidant system to reduce food-induced oxidative stress [59] and (ii) the fact that the regular diet contains carbohydrates, lipids, and proteins, as well as minerals and trace elements that boost antioxidant capacity [11,55,56]. The current study does admit to several limitations. A small sample size is the first issue. Second, we only included younger people in our research; we did not include people of any particular age. It is widely recognized that oxidative stress is impacted by age [60,61,62]. Researching people of different ages might yield different findings. Third, our observation time after bar consumption was only 3 h, which may need to be extended. This research will be a piece of base evidence on the impact of formulated high-protein and high-energy bars on oxidative stress.

## 5. Conclusions

The present study examined the postprandial antioxidant response to DBBs or FBBs in healthy individuals. Practically, FBBs were significantly higher in free PC than DBBs, while DBBs were significantly higher in bound PC than FBBs. Twenty healthy participants were fed 140 g (510 Kcal) of DBB and FBB, and then the TPC, T-AOC, MDA, and SOD in the plasma of the healthy individuals were monitored for 3 h. Postprandial TPC peaked after 120 min. DBBs increased T-AOC content significantly after 120 and 180 min, while FBBs did not. MDA levels decreased significantly 180 min after DBB ingestion, but not after FBB ingestion. SOD responded to DBBs significantly, while no significant response was noted up to 3 h after consuming FBBs. Relative changes in terms of the AUC values calculated only exuded a significant effect for TPC and SOD plasma concentrations but not for TAOC or MDA. Pearson’s correlation coefficients showed strong links between analyzing times and TPC, T-AOC, and MDA. A strong positive relationship (*p* < 0.01) was established between T-AOC and MDA or SOD, as well as between both of them. The principal component analysis showed that PCs 1 (75.88%) and 2 (18.58%) had the maximum variability with 94.45% cumulative variance. SOD and TPC were positively associated at 60 and 120 min after DBB consumption, while MAD was negatively correlated. In conclusion, this research will provide evidence of the impact of formulated high-protein and high-energy bars on oxidative stress. Expanding the experimental time in future investigations will be necessary to validate the present results.

## Figures and Tables

**Table 1:** Free and bound phenolic contents in DBBs and FBBs.

Bars	Free PC [mg GAE g^−1^]	Bound PC [mg GAE g^−1^]
DBBs	10.15 ± 1.61 ^b^	6.19 ± 0.99 ^a^
FBBs	12.98 ± 1.23 ^a^	3.57 ± 0.83 ^b^

Free PC: free polyphenol content; Bound PC: bound polyphenol content; ^a,b^: within the same column, there is no significant difference (*p* > 0.05) between any two means with the same superscripted letters; data are presented as mean ± SE, *n* = 3.

**Table 2:** General and anthropometric characteristics of study participants (mean ± SE), *n* = 20.

Age (years)	21.4 ± 0.9
Height (cm)	170.0 ± 1.3
Body weight (kg)	66.3 ± 1.7
BMI (kg m^−2^)	22.9 ± 0.4
Body fat mass (kg)	14.5 ± 1.1
Total body water	37.9 ± 0.9
Body fat-free mass (kg)	29.2 ± 0.8

**Table 3:** Pearson’s correlation coefficients of postprandial oxidative stress biomarkers in the blood of healthy individuals upon consuming FBBs or DBBs.

		TPC	T-AOC	MDA	SOD
Sampling time	Pearson’s correlation	0.183 *	0.221 **	−0.163 *	0.008
	Sig. (2-tailed)	0.021	0.005	0.040	0.915
TPC	Pearson’s correlation		−0.095	−0.113	−0.073
	Sig. (2-tailed)		0.234	0.155	0.357
T-AOC	Pearson’s correlation			0.279 **	0.709 **
	Sig. (2-tailed)			0.000	0.000
MDA	Pearson’s correlation				0.298 **
	Sig. (2-tailed)				0.000

*: correlation is significant at the *p* = 0.05 level (2-tailed); **: correlation is significant at the *p* = 0.01 level (2-tailed); *n* = 20 (×2 experimental trials in cross-over manner).

## Data Availability

Data are contained within the article.
